# Analysis of Wi-SUN FAN Network Formation Time

**DOI:** 10.3390/s24041142

**Published:** 2024-02-09

**Authors:** Ananias Ambrosio Quispe, Rodrigo Jardim Riella, Luciana Michelotto Iantorno, Leonardo Santanna Mariani, Evelio M. Garcia Fernandez

**Affiliations:** 1 Department of Electrical Engineering, Federal University of Paraná—UFPR, Curitiba 81531-980, Brazil; riella@lactec.com.br (R.J.R.); evelio@ufpr.br (E.M.G.F.); 2 Electronic and Digital Systems, Lactec Institutes, Curitiba 80215-090, Brazil; luciana.iantorno@lactec.com.br (L.M.I.); leonardo.mariani@lactec.com.br (L.S.M.)

**Keywords:** Wi-SUN FAN, simulator, node connection process, Contiki-NG

## Abstract

The Wi-SUN FAN (Wireless Smart Ubiquitous Network Field Area Network) standard is attracting great interest in various applications such as smart meters, smart cities and Internet of Things (IoT) devices due to the attractive features that the standard offers, such as multihop and mesh topologies, a relatively high data rate, frequency hopping, and interoperability between manufacturers. However, the process of connecting nodes in Wi-SUN FAN networks, which includes discovering, joining, and forming the network, has been shown to be slow, especially in multihop environments, which has motivated research and experimentation to analyze this process. In the existing literature, to measure network formation time, some authors have performed experiments with up to 100 devices, which is a costly and time-consuming methodology. Others have used simulation tools that are difficult to replicate, because little information is available about the methodology used or because they are proprietary. Despite these efforts, there is still a lack of information to adequately assess the formation time of Wi-SUN FAN networks, since the experimental tests reported in the literature are expensive and time-consuming. Therefore, alternatives such as computer simulation have been explored to speed up performance analysis in different scenarios. With this perspective, this paper is focused on the implementation of the Wi-SUN FAN network formation process using the Contiki-NG open source operating system and the Cooja simultor, where a functionality was added that makes it possible to efficiently analyze the network performance, thereby facilitating the implementation of new techniques to reduce network training time. The simulation tool was integrated into Contiki-NG and has been used to estimate the network formation times in various indoor environments. The correspondence between the experimental and numerical results obtained shows that our proposal is efficient to study the formation process of this type of networks.

## 1. Introduction

Wi-SUN FAN, an open protocol-based wireless communication standard for low-power and lossy networks (LLNs) [1], is gaining attention in various applications, such as smart meters, smart cities, and the Internet of Things (IoT) [2], where thousands of nodes are interconnected [3,4]. The standard, part of the Wi-SUN Alliance [5], is characterized by its mesh network configuration with multihop communication utilizing the RPL protocol [6,7] that enables dynamic network formation, thereby offering higher data rates, frequency hopping to prevent interference, and interoperability among different manufacturers [1]. However, certain security aspects need flexibility on the provider’s side [8], and the ecosystems for upper layers are yet to be defined [9].

Special attention must be given to the process of connecting nodes to the network, which encompasses node discovery, merging, and network formation. Previous experimental work [10] revealed slow connection times in multihop networks, thus prompting investigations into the parameter settings of the trickle timer algorithm [11]. While Wi-SUN FAN documentation provides parameter recommendations [1] for small and large networks, experimental evaluations [12] indicate persistently high connection times, especially in large network configurations.

To achieve the full performance predicted by the standard, some device manufacturers and other researchers have conducted experiments in controlled environments with up to 100 devices [12,13,14]. These experiments emphasize the importance of configuring parameters based on network size to balance connection time, latency, and device scalability. Other works involving a smaller number of devices, such as those in [15], conducted performance evaluations in an urban setting, thus utilizing a maximum of three devices. The findings affirmed commendable performance in terms of packet success rate and latency within an urban scenario. Furthermore, [16] assessed the data flow in various advanced metering infrastructure (AMI) applications using a maximum of seven devices, thus affirming the Wi-SUN FAN’s capability to effectively support diverse intelligent network applications.

Based on the results above, it can be seen that the information available for evaluating the performance of the Wi-SUN FAN is still insufficient, since the application of an experimental methodology for this purpose is time-consuming, and its cost increases with the number of devices. Consequently, other alternatives such as computer simulation are needed to reproduce the behavior of the standard in order to allow for a quick performance analysis in different scenarios and with different parameterizations.

With this perspective, some works were published that were focused on the development of simulators and emulators to evaluate the performance of the Wi-SUN FAN. For instance, in [12], Silicon Labs presented simulation results using the stack already developed for their devices and created internal tools that run multiple instances of the Wi-SUN FAN stack for the simulated nodes; therefore, this simulator is confidential. Additionally, in [17,18], emulators were proposed in order to address the performance of the Wi-SUN FAN at the physical layer, with the results obtained being focused on the transmission characteristics in large-scale networks; therefore, these emulators are not suitable for implementing new algorithms in other layers of the standard.

In this paper, the process of joining nodes to Wi-SUN FAN networks is implemented using Contiki-NG, an open source operating system (OS) that is compatible with a wide range of wireless sensor network hardware, which provides protocols libraries for smart devices [19] in low-power networks that can be configured and tested using the Cooja simulator for different network scenarios. In this way, different available protocols [20,21] that have been used to facilitate the implementation of the Wi-SUN FAN standard can be leveraged, in particular the RPL protocol that is used for network formation and allows for the implementation of the node connection process that goes through the join states in the Wi-SUN FAN [1].

The implementation of the standard is done mainly in the medium access control (MAC) layer of the Contiki-NG stack, where two main processes are configured, such as the node discovery and joining process, using the MLME-WS-ASYNC-FRAME technique [1] and frequency hopping procedures. These procedures enable the implementation of asynchronous, unicast, and broadcast communication of the Wi-SUN FAN standard. In this sense, we added a feature to Contiki-NG that allows for a quick performance evaluation of Wi-SUN FAN network scenarios, thereby providing easy scalability of the number of nodes in different network topologies and allowing the use of fewer resources compared to experimental work.

The main objective of this work is to develop a simulation tool that allows for evaluating, from a temporal point of view, the connection process of the nodes in Wi-SUN FAN networks. In particular, regarding the long connection time of the Wi-SUN FAN nodes, the functionality being proposed will make it possible to quickly simulate the process of discovering, joining, and forming the network, which will facilitate the implementation of new techniques to reduce the network formation time. Thus, our main contribution is the following:

- The incorporation of the simulation tool developed within the Contiki-NG OS, which allowed for calculating and validating the formation times of Wi-SUN FAN networks through experiments in indoor environments.

The remainder of this paper is structured as follows. Section 2 describes some technical background information about Wi-SUN FAN networks. Section 3 describes the structure of the simulator. In Section 4, the proposed solution is validated for each scenario studied, while Section 5 presents the results. Finally, Section 6 concludes the paper.

## 2. Background Information

**Figure 1:**
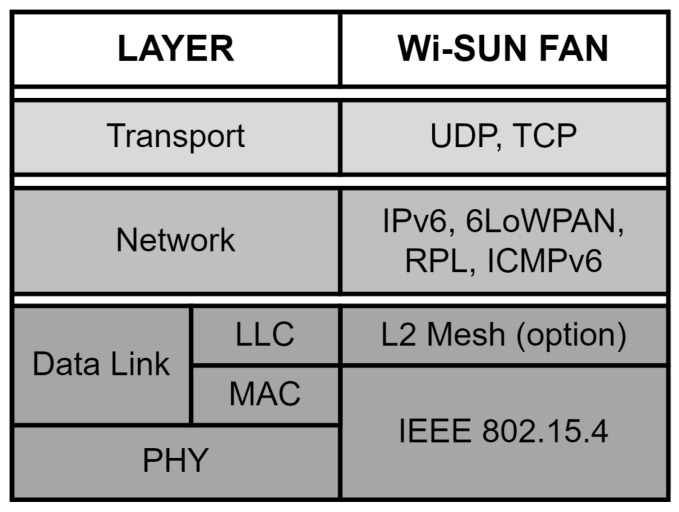
Wi-SUN FAN profile.

The Wi-SUN FAN is a standard that belongs to the Wi-SUN alliance [22] solution profile list and is intended for various applications for large-scale outdoor networks. Figure 1 shows the different layers that make up the standard [1], where the physical layer is based on the IEEE802.15.4 [23] protocol, and the frequency band used is regulated according to the region of operation. Regarding the data link layer, the MAC sublayer is defined in the IEEE802.15.4 protocol and uses frequency hopping, and the LLC (logic link control) sublayer uses the optional L2 mesh service. At the network layer, the IPv6 over low-power wireless PAN (6LoWPAN) adaptation is used for the network service, and for the network formation, it uses the RPL protocol, where the Internet Control Message Protocol for IPv6 (ICMPv6) is used for its control packets. Finally, the transport layer supports the User Datagram Protocol (UDP) and Transmission Control Protocol (TCP) packet traffic. The upper layers of the OSI model (application, presentation, and session) are not part of the Wi-SUN FAN protocol stack.

A Wi-SUN FAN network is hierarchical and consists of three types of nodes: a (a) border router (BR), which manages all source routing information for the other nodes; a (b) router (R), which is the node that handles the upward and downward forwarding of packets and maintains the routing tables for its neighboring nodes; and a (c) leaf (F), which is the node that provides the minimum capabilities for discovering and entering the PAN, as well as sending and receiving packets.

### 2.1. Frequency Hopping

**Figure 2:**
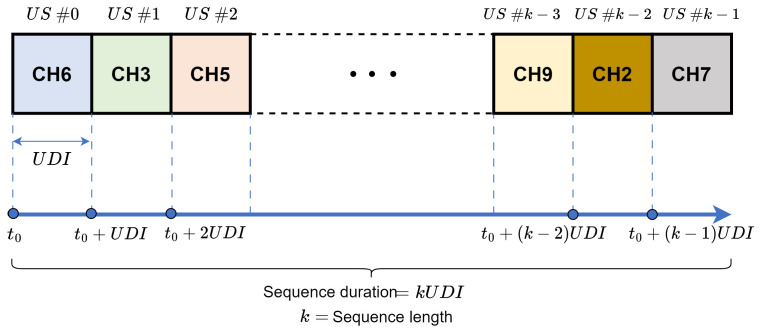
Unicast frequency hopping.

The nodes support channel hopping over a pseudorandom sequence defined by the channel function for unicast and broadcast transmissions [1,24,25,26,27]. Unicast frequency hopping is receiver-directed, with the unicast hopping sequence derived from the node’s MAC address and the set of available channels [13]. Figure 2 shows the unicast frequency hopping, where the hopping sequence can be thought of as a repeating sequence of a UDI or unicast slot (US), where the US number identifies a specific location in the sequence.

**Figure 3:**
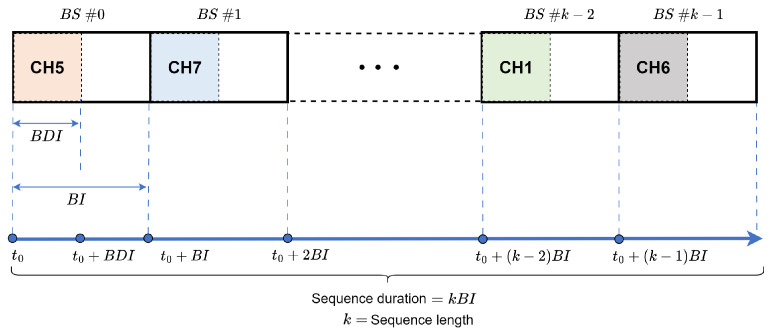
Broadcast frequency hopping.

Figure 3 shows the broadcast frequency hopping, where the hopping sequence can be thought of as a repeating sequence of broadcast intervals (BI) or broadcast slots (BS), with the BS number denoting a specific location in the sequence. The broadcast dwell interval (BDI) is usually much shorter than the BI.

### 2.2. Discovery and Joining of Nodes

**Figure 4:**
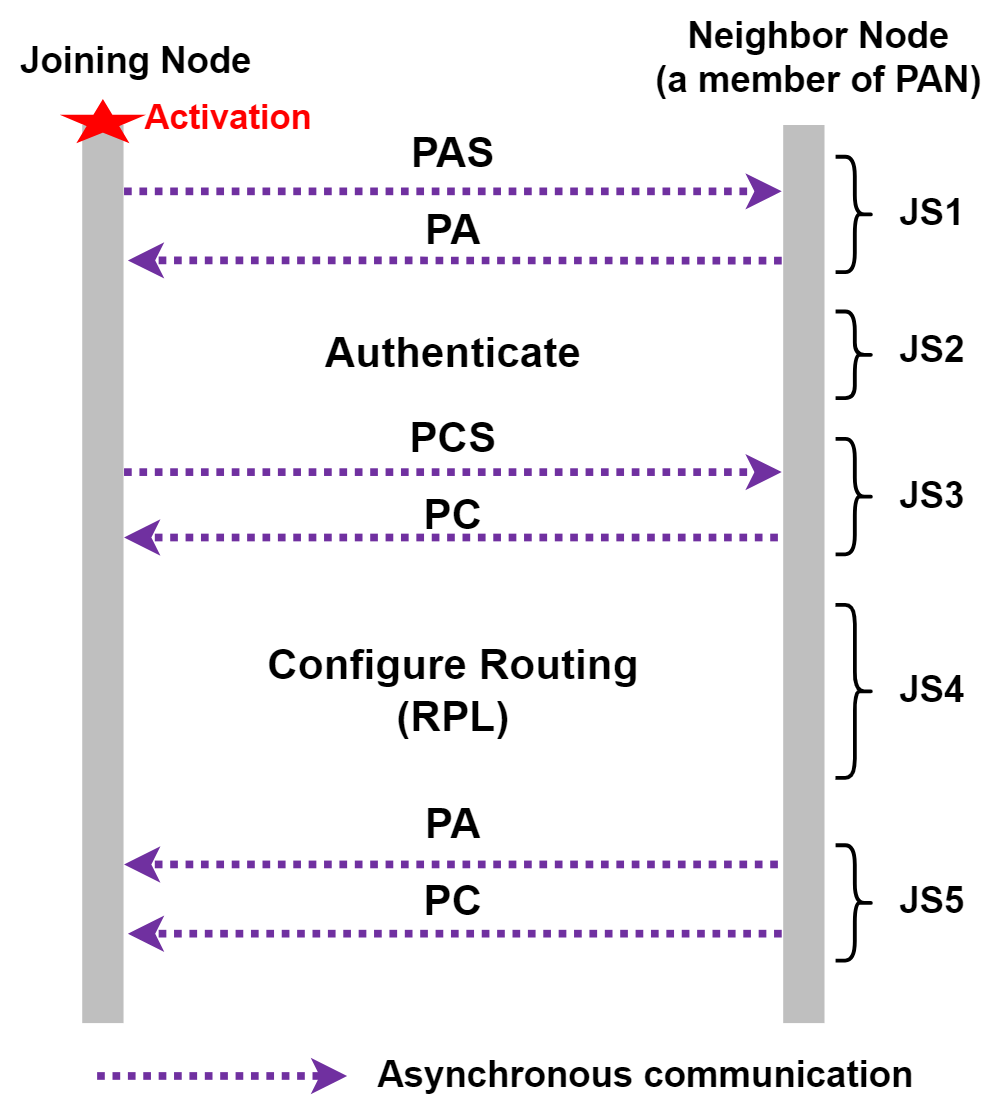
Discovery and joining process.

An RB and at least one R or F node are required to establish a Wi-SUN FAN network. Any node wishing to join a PAN must go through five join states [1,10,24]: (i) Join State 1 (JS1)—the discovery process of available PANs and asynchronous communication through the PA (PAN Advertisement) and PAS (PAN Advertisement Solicit) messages; (ii) Join State 2 (JS2)—the node authentication process (in this work, this process was not implemented in this simulator due to its high complexity); (iii) Join State 3 (JS3)—the PAN configuration data collection process and asynchronous communication through the PC (PAN Configuration) and PCS (PAN Configuration Solicit) messages; (iv) Join State 4 (JS4)—the node routing configuration process using the RPL protocol; and (v) Join State 5 (JS5)—the node is then part of the Wi-SUN FAN network. Figure 4 shows the discovery and joining process in a simplified form, thereby mainly showing the exchange of messages between JS1 and JS3.

When a node is discovered, the Wi-SUN FAN uses the MLME-WS-ASYNC-FRAME mechanism to transmit association messages, which is a service described in [1] that is an equivalent to the MLME (MAC sublayer management entity) services [23]. This process starts in JS1, where an RB or other R nodes already connected to the network announce the existence of the network by periodically broadcasting PA messages with the minimum parameters for a new node so that the new node can choose from several available PANs.

**Figure 5:**
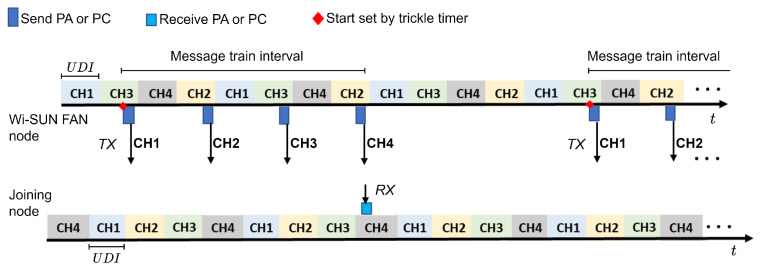
Process of sending or receiving PA or PC packets that allow for the synchronization of new nodes.

Figure 5 shows an example with an announcement node and a joining node that start listening to a sequence of four channels. Note that the sequence of channels is different for each node and that they start at different times. The dwell time on each channel is defined by the variable UDI, and the number of PA messages is determined by the number of available channels. The packets are sent sequentially according to the list of available channels, starting with the first one (in our example, from CH1 to CH4). Once the sequence of PA messages is over, it waits until the next start time, which is determined by the trickle timer. In this example, the joining node receives the PA packet on CH4.

**Figure 6:**
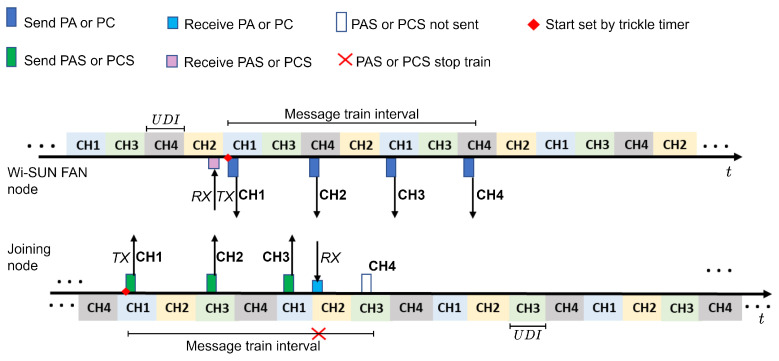
Process of sending and receiving PAS or PCS packets that allows nodes to throttle the rate at which their neighbors transmit PA or Ps packets.

In addition, joining nodes can force the PA to be sent by its neighbors (before the time designated by the trickle timer) by sending PAS messages. Figure 6 illustrates the process of sending the PAS message train to speed up the sending of PA messages. When the advertising node receives a PAS, it triggers the start of the PA message train. The joining node that receives a PA interrupts this process when it is in the process of sending a PAS message train.

In JS3, PC messages are transmitted in encrypted form when a wide network configuration is shared. As in the PA, PC transmission is controlled by the trickle timer as shown in Figure 5, and the joining node can force transmission (before the time scheduled by the trickle timer) by transmitting PCS messages as shown in Figure 6.

Therefore, this node association process is governed by the trickle timer algorithm [11], which defines the time intervals for sending these control messages (PA, PAS, PC, PCS). The Wi-SUN FAN documentation shows configuration recommendations on this algorithm, thereby defining mainly two configurations: small and large networks. In [12], manufacturers consider a network with 1 to 100 devices to be small, while networks with more than 800 devices are considered large networks. They also consider a configuration of medium networks when the number of devices is between 100 and 800. In the association process, these configurations have an important impact on the relationship between the connection time, latency, and scalability of the network. A consideration to take into account with the Wi-SUN FAN standard is to choose the type of configuration according to the type of application.

## 3. Implementing the Wi-SUN FAN Node Association Procedure in Contiki-NG

In this section, we describe the implementation, within the Contiki-NG stack structure, of a functionality that allows for evaluating the process of connecting nodes to Wi-SUN FAN networks.

**Figure 7:**
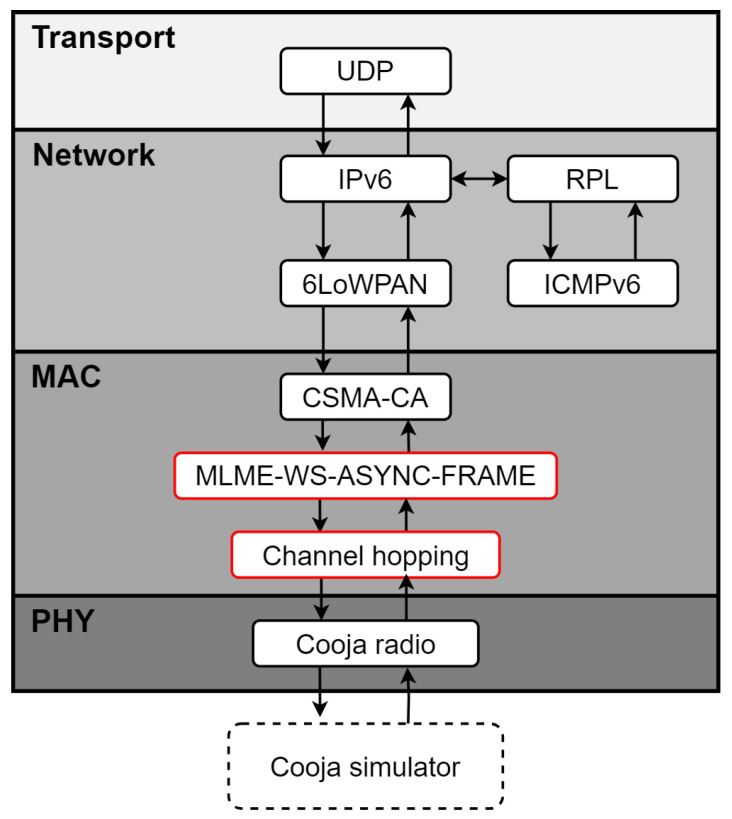
Contiki-NG stack with new functionality incorporated.

Figure 7 shows the added mechanisms, where frequency hopping and the MLME-WS-ASYNC-FRAME association mechanism are added to the MAC layer. Unlike Figure 1, the order of use of the protocols using the Cooja simulator is shown. On the other hand, the CSMA-CA (carrier-sense multiple access with collision avoidance) and RPL files were adapted to take into account the frequency hopping process of the Wi-SUN FAN.

The main reason for using the Cooja simulator is that it has allowed us to replicate the scenarios used in the experiments, where each node is a compiled and executed Contiki system. In addition, it has an interface to analyze and interact with the nodes; this makes it possible to capture messages from the simulations and facilitates the visualization of the network. It is also possible to create custom scenarios [28].

The channel hopping functionality is implemented according to the technical information described in Section 2.1; this functionality is added to a folder called ‘wisun-mac/’, which is located inside the ‘net/’ folder of the Contiki-NG stack. The MLME-WS-ASYNC-FRAME association functionality is implemented as described in Section 2.2, which is placed in the same folder as the previous functionality. For the nodes to follow the association process of a Wi-SUN FAN network of Figure 4, two types of nodes with BR and R characteristics are generated; in the simulator, they are the files “border-router.c” and “router.c”.

The association process of these nodes with these two functionalities are implemented in the Algorithm 1. A node performs a channel scan call (CHscan) by listening to the PA and PC packets on the available channels in the *V* (the vector containing the channels available for the network), one by one in the order of their occurrence in *V*, using a round-robin procedure, thus starting on a random channel given by a channel function.

The nodes initialize the following variables: the number of channels available (Nch), which can be used to scale the number of channels for *V*; the channel index ($I_{c}$), which is used to select the channel within *V*; and the channel scan time (UDIscan), which sets the scan time for each channel (lines 1–3 in Algorithm 1). The vector *V* is generated by the channel function, and $I_{c}$ is set to the value 0 to start a scan in the first channel of the vector *V* (lines 4–5 in Algorithm 1).

When the nodes are in JS1 and want to connect, they listen to each channel for a period of time specified by the UDIscan. Nodes select a channel present in the *V* each time they change the currently scanned channel using the round-robin strategy; that is, $I_{c}$ increases by 1 (lines 6–13 in Algorithm 1). As soon as the unassociated node hears a PA, it extracts the unicast communication timing information from the Wi-SUN FAN node and, as JS2 was not implemented in the simulator, the node jumps to JS3 (lines 14–16 in Algorithm 1).

In JS3, the node performs the same process as before by listening to each channel of the *V* for a period of time specified by the UDIscan and performing a round-robin scan (lines 19–25 in Algorithm 1). Once the nonassociated node listens to a PC, it extracts the information from the broadcast communication to get to JS4 (lines 26–28 in Algorithm 1).
**Algorithm 1** Wi-SUN FAN association1:Nch: number of available channels2:$I_{c}$: channel index3:UDIscan: channel scan time4:V[] ← channel_function()5:$I_{c}$ ← 06:**if** node = JS1 **then**7:    $CHscan\leftarrow V[I_{c}]$8:    **while** is not associated **do**9:        **if** the scan time has expired **then**10:           resets the scan time11:           $I_{c}\leftarrow \left(I_{c}+1\right)$12:           $CHscan\leftarrow V[I_{c}]$13:        **end if**14:        **if** PA is received **then**15:           node ← JS316:        **end if**17:    **end while**18:**end if**19:**if** node = JS3 **then**20:    **while** is not associated **do**21:        **if** the scan time has expired **then**22:           resets the scan time23:           $I_{c}\leftarrow \left(I_{c}+1\right)$24:           $CHscan\leftarrow V[I_{c}]$25:        **end if**26:        **if** PC is received **then**27:           node← JS428:        **end if**29:    **end while**30:**end if**

At the end of the process described in Algorithm 1, the Contiki-NG functionalities are used. The CSMA-CA protocol for channel access and the RPL protocol for network formation are used for JS4. Finally, the node enters JS5.

## 4. Validation

In order to validate the proposed functionality to investigate the connection time of the nodes, several experiments were carried out in an indoor laboratory environment and then compared with Cooja simulations using common topologies. Therefore, three network topologies were defined with a small number of nodes using a similar methodology as that in [29], which facilitated the analysis.

**Figure 8:**
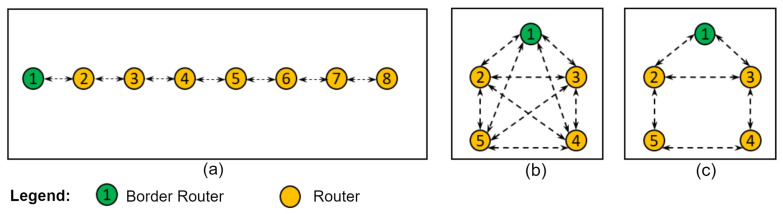
Network topologies. (**a**) Linear. (**b**) Fully connected. (**c**) Mesh scheme.

Figure 8a shows a linear topology consisting of one BR node and seven R nodes, thus confirming a linear interaction between the nodes. Figure 8b,c show the fully connected and mesh topologies, respectively, consisting of one BR node and four R nodes. The interaction in the fully connected topology is that the nodes can receive/transmit directly from/to any node in the network. The interaction in the mesh topology is that the nodes can have more than two neighbors but cannot transmit/receive to/from other nodes in the network, either due to a range restriction or an enforced configuration that restricts communication with certain neighbors.

### 4.1. Experiment Setup

For the experiments, a procedure similar to that in presented in [30] was followed for the three topologies, where the experiments were conducted using Wi-SUN FAN devices in the Lactec Electronics Laboratory in a controlled environment for device interaction. As JS2 was not implemented in our proposal, in the experiments, the connection time of the network was calculated by measuring the times in JS1, JS3, and JS4. The messages from the devices indicating the completion of the states are shown in Table 1.

**Figure 9:**
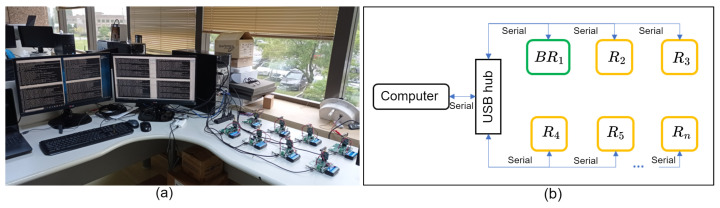
Experimental scenario. (**a**) Testbed. (**b**) Connection scheme.

To record the network connection times in an experimental scenario, a testbed was implemented as shown in Figure 9a, where the messages in Table 1 were recorded for each device. The device connection scheme is shown in Figure 9b.

The devices were configured with authentication disabled; the minimum distance between them was about 10 cm and to achieve the configurations of the topologies between the devices in the Figure 8. A whitelist was used, which is an address filter in the software that makes it possible to limit the RF (radio frequency) communication between the devices and to define their neighbors. Table 2 shows the neighbors defined for each topology.

The parameters configured in the devices for the three topologies are shown in Table 3.

Each experiment was repeated 10 times for each topology. The procedure for each experiment was to connect the R-type nodes first in an ordered manner, and lastly connect the RB node. Measurements were made in the connection states of each node according to the configured topology. As the sample was small in each experiment, the Student’s t distribution was used to calculate the confidence interval that is shown in the figures in Section 5.

In addition, a 20-node random mesh topology was experimentally verified using the testbed and Table 4 for the neighborhood configuration of each node.

### 4.2. Simulator Setup

**Figure 10:**
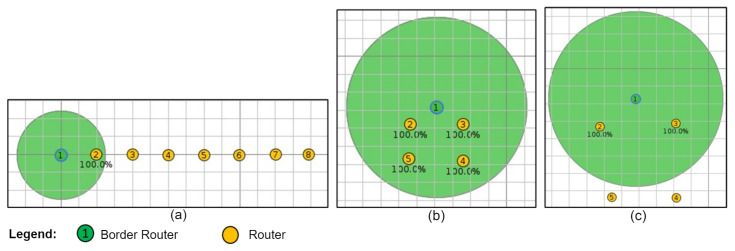
Topologies configured in Cooja: (**a**) Linear. (**b**) Fully connected. (**c**) Mesh.

The topologies implemented in the experiments were replicated in the simulator. Figure 10 shows the linear, fully connected, and mesh topologies configured in the Cooja simulator; to define the neighbors of each node in the simulator, Table 2 was used.

Each simulation was repeated 10 times for each topology, thereby changing the activation time of the nodes randomly in each simulation. In all simulations, the messages of the transition process of the JSs shown in Table 1 were recorded. The parameters configured in the simulator are shown in Table 5.

**Figure 11:**
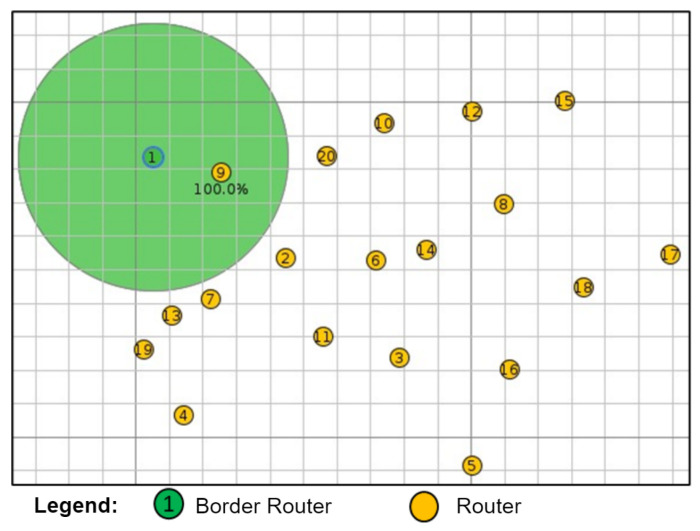
Network with 20 nodes.

To validate the simulator with the 20-node mesh topology experiment, Cooja was used to create a network with a random distribution of one RB and nineteen R nodes, as shown in Figure 11.

**Figure 12:**
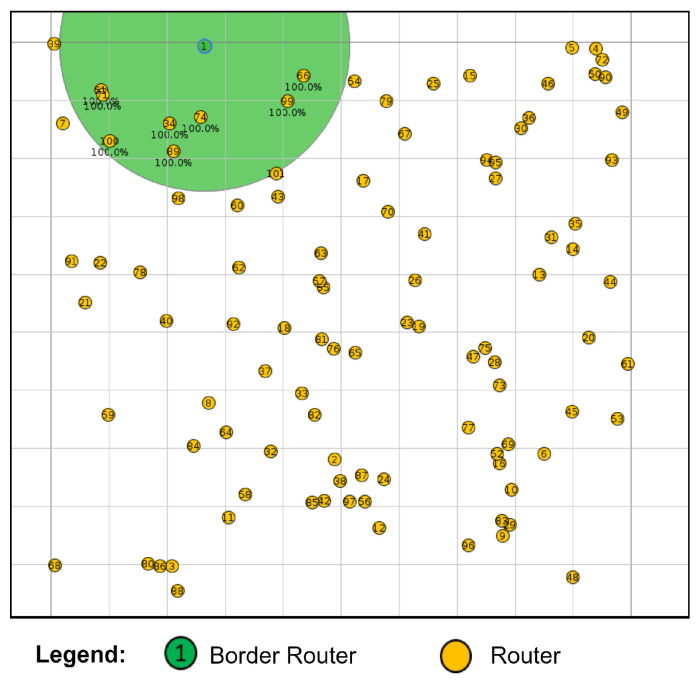
Network with 101 nodes.

In addition, a mesh network consisting of one BR and one hundred R was generated for comparison with the result presented in [12], as shown in Figure 12.

## 5. Results

The experiments and simulations in this study include data from the indoor laboratory. In addition, simulations were performed to compare the network connection times with the experimental results presented in [12], where 100 devices were used in small-, medium-, and large-scale configurations. Next, the results obtained in the indoor laboratory for the linear, fully connected, and mesh topologies are shown.

### 5.1. Linear Topology

**Figure 13:**
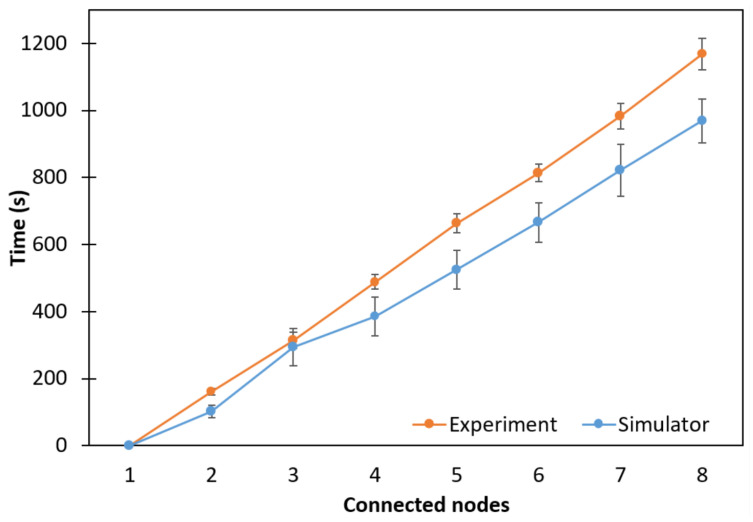
Network formation time considering a linear topology.

The connection time of the nodes in the linear topology is shown in Figure 13, where average network formation times of 1167.6 s and 969 s were observed for the experimental and simulation results, respectively. Node 1 is the RB starting at JS5, with the network connection time measurements starting at node 2 (node R). Since this is a linear topology, node 2, which is closest to the RB, will connect first. Node 3 must wait until node 2 has finished its connection process so that it can start its own connection process. This happens sequentially for the other nodes until the last R node joins the network. This process is called a multihop connection.

With this linear topology, it was possible to verify that each node R used a similar connection time for each hop in the network. The simulation curve followed the same trend as the experiment curve, namely constant growth.

### 5.2. Fully Connected Topology

**Figure 14:**
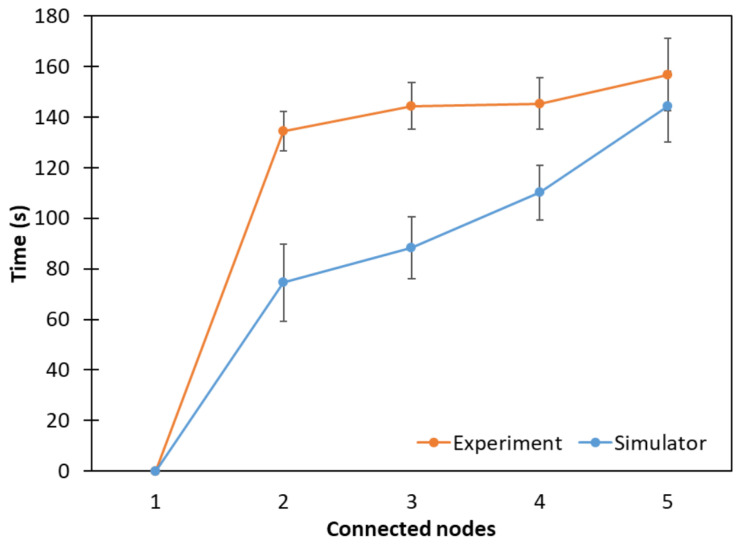
Network connection time considering a fully connected topology.

The connection time of the nodes in the fully connected topology is shown in Figure 14, where average network formation times of 156.8 s and 144.21 s were computed from the experimental and simulation results, respectively. The RB is node 1, which was already connected. In Figure 14, it can be seen that the first nodes in the simulation connected faster compared to the experiments, which is due to the ideal and controlled environment of the simulation. However, it can be observed that the simulation results followed a growth trend similar to that of the experimental results.

### 5.3. Mesh Topology

**Figure 15:**
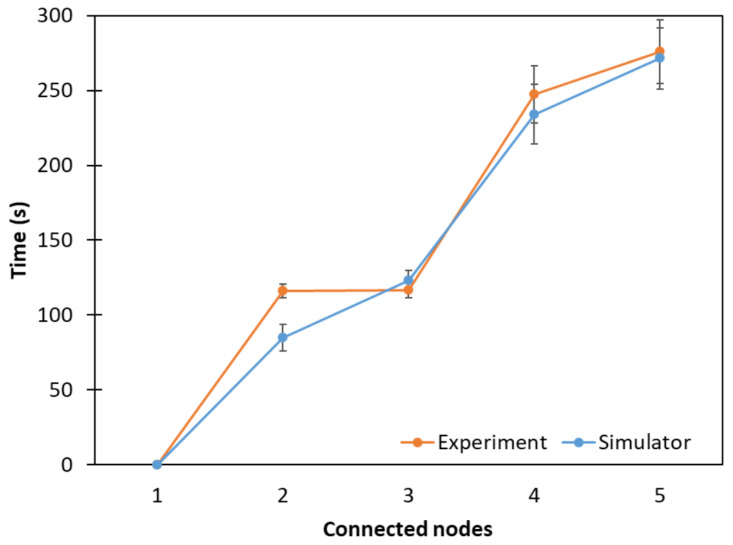
Network connection time considering a mesh topology.

Figure 15 shows the connection time of the nodes in the mesh topology, where average network formation times of 276 s and 271.42 s for the experimental and simulation results, respectively, were observed. As in the previous topologies, node 1 represents the RB that is already connected to JS5. It can be seen that the experimental and simulation results were very close to each other.

**Figure 16:**
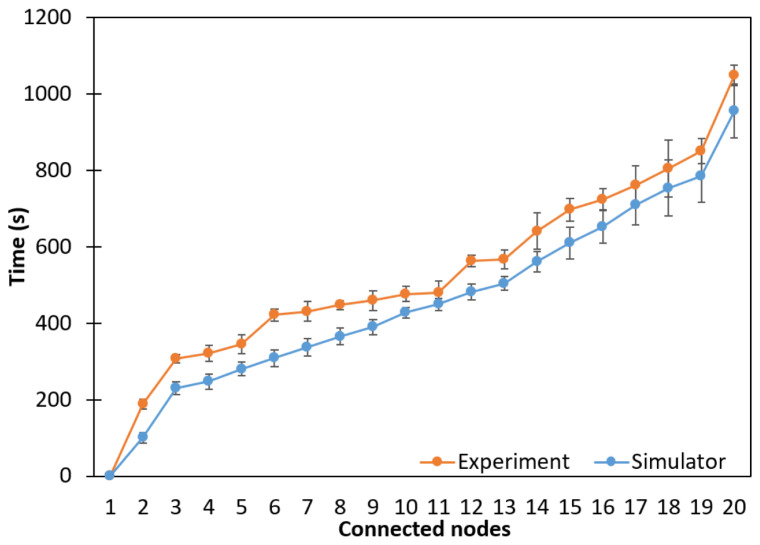
Network connection time considering a 20-node mesh topology.

To verify the behavior of the simulated mesh topology in a larger scenario, a network with twenty nodes (1 BR node and 19 R nodes) was tested. The total connection times of all nodes for the experiments and the simulations were 1049.50 s and 955.35 s, respectively. A similar behavior can be seen in both curves that coincide for some connection time values, mainly in the last connected nodes, as shown in Figure 16.

**Figure 17:**
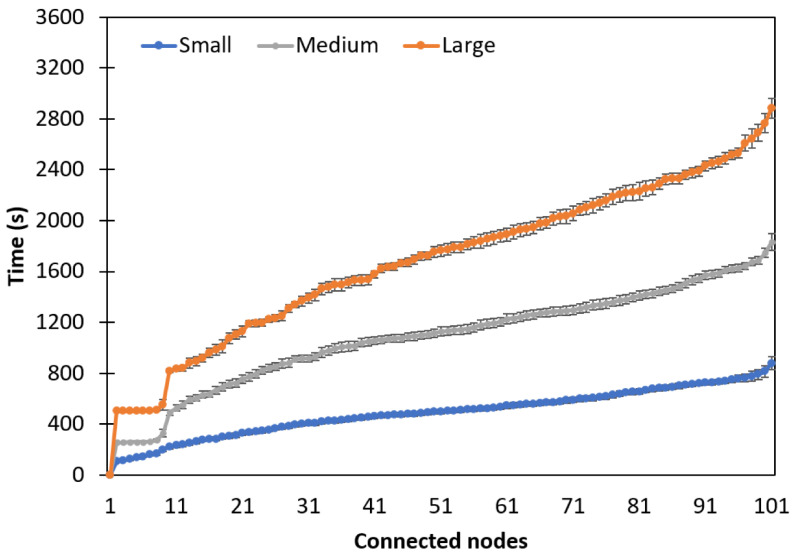
Network connection time using configurations for small-, medium-, and large-scale networks.

Aiming at comparison with experimental results reported in another work, we performed simulations using the same configurations as those used in [12], where the connection time was measured in a network with 100 nodes. For this, small, medium, and large network sizes were considered in the trickle timer algorithm to configure the generation times of the control packets that flow in the network and that impact the scalability of the network. Figure 17 shows the simulation results where the impact on connection time of each type of network configuration can be observed. In particular, for the network with 100 nodes, the connection times obtained were very similar to those measured in [12], where connection times of 13, 37, and 55 min were measured for small, medium, and large network configurations, respectively.

## 6. Conclusions

In this paper, we introduced a simulation tool to analyze the process of joining nodes to Wi-SUN FAN networks by implementing a new procedure in the MAC layer of the Contiki-NG stack that made it possible to measure the network formation time in this standard. Through a series of experiments in indoor environments, we observed a coincident trend between the connection times measured in the experiments and the simulated ones, which was more evident in the mesh topology. Simulations were also carried out replicating experimental scenarios reported in other work, which allowed us to verify similar results regarding the connection times of nodes to Wi-SUN FAN networks. All this provides strong support for the adoption of the proposed functionality as a valuable tool to study the node formation process in Wi-SUN FAN networks.

As future work, the developed functionality will be used to reduce the network formation time through the design of parallel rendezvous algorithms, which would take advantage of the PAS and/or PCS packets to generate clusters of presynchronized nodes in the association process, which is a widely used strategy in cognitive radio networks.

## Figures and Tables

**Table 1:** JS completion indicator.

State	Completion Indicator
JS1	Receipt of PA message
JS3	Receipt of PC package
JS4	Receives last message from DAO (destination advertisement object) routing process and enters JS5

**Table 2:** Neighbors for each topology.

Device	Linear	Fully Connected	Mesh
$BR_{1}$	$R_{2}$	$R_{2}$, $R_{3}$, $R_{4}$, $R_{5}$	$R_{2}$, $R_{3}$
$R_{2}$	$BR_{1}$, $R_{3}$	$RB_{1}$, $R_{3}$, $R_{4}$, $R_{5}$	$RB_{1}$, $R_{3}$, $R_{5}$
$R_{3}$	$R_{2}$, $R_{4}$	$RB_{1}$, $R_{2}$, $R_{4}$, $R_{5}$	$RB_{1}$, $R_{2}$, $R_{4}$
$R_{4}$	$R_{3}$, $R_{5}$	$RB_{1}$, $R_{2}$, $R_{3}$, $R_{5}$	$R_{3}$, $R_{5}$
$R_{5}$	$R_{4}$, $R_{6}$	$RB_{1}$, $R_{2}$, $R_{3}$, $R_{4}$	$R_{2}$, $R_{4}$
$R_{6}$	$R_{5}$, $R_{7}$	-	-
$R_{7}$	$R_{6}$, $R_{8}$	-	-
$R_{8}$	$R_{7}$	-	-

**Table 3:** Configuration parameters.

Parameter	Description
Frequency range	902–907.5 MHz and 915–928 MHz (Brazil)
Operation mode	1b
Data rate	50 kbps
Transmission power	10 mW
Frequency hopping	Enabled
Number of channels	90

**Table 4:** Neighbors of the nodes.

Device	Neighbors	Device	Neighbors
$BR_{1}$	$R_{9}$	$R_{11}$	$R_{2}$, $R_{3}$, $R_{6}$, $R_{7}$
$R_{2}$	$R_{6}$, $R_{7}$, $R_{9}$, $R_{11}$, $R_{13}$, $R_{20}$	$R_{12}$	$R_{8}$, $R_{10}$, $R_{15}$
$R_{3}$	$R_{5}$, $R_{6}$, $R_{11}$, $R_{14}$, $R_{16}$	$R_{13}$	$R_{2}$, $R_{4}$, $R_{7}$, $R_{19}$
$R_{4}$	$R_{7}$, $R_{13}$, $R_{19}$	$R_{14}$	$R_{3}$, $R_{6}$, $R_{8}$, $R_{10}$
$R_{5}$	$R_{3}$, $R_{16}$	$R_{15}$	$R_{8}$, $R_{12}$
$R_{6}$	$R_{2}$, $R_{3}$, $R_{11}$, $R_{14}$, $R_{20}$	$R_{16}$	$R_{3}$, $R_{5}$, $R_{18}$
$R_{7}$	$R_{2}$, $R_{4}$, $R_{9}$, $R_{11}$, $R_{13}$, $R_{19}$	$R_{17}$	$R_{18}$
$R_{8}$	$R_{12}$, $R_{14}$, $R_{15}$, $R_{16}$	$R_{18}$	$R_{8}$, $R_{16}$, $R_{17}$
$R_{9}$	$BR_{1}$, $R_{2}$, $R_{7}$, $R_{20}$	$R_{19}$	$R_{4}$, $R_{7}$, $R_{13}$
$R_{10}$	$R_{12}$, $R_{14}$, $R_{20}$	$R_{20}$	$R_{2}$, $R_{6}$, $R_{9}$, $R_{10}$

**Table 5:** Parameters for configuring the simulator.

Parameter	Description
UDI	100 ms
BDI	400 ms
BI	1600 ms
Number of channels	90
Network type	Small

## Data Availability

Data are contained within the article.

## Abbreviations

The following abbreviations are used in this manuscript:

BDI	Broadcast Dwell Interval
BI	Broadcast Intervals
BS	Broadcast Slots
JS	Join State
L	Leaf
LLN	Low-Power and Lossy Network
OS	Operating System
PA	PAN Advertisement
PAN	Personal Area Networks
PAS	PAN Advertisement Solicit
PC	PAN Configuration
PCS	PAN Configuration Solicit
R	Router
RPL	IPv6 Routing Protocol for Low-Power and Lossy Networks
UDI	Unicast Dwell Interval
UDP	User Datagram Protocol
US	Unicast Slots
Wi-SUN FAN	Wireless Smart Ubiquitous Network Field Area Network
